# Cardiac metabolism in a new rat model of type 2 diabetes using high-fat diet with low dose streptozotocin

**DOI:** 10.1186/1475-2840-12-136

**Published:** 2013-09-24

**Authors:** Latt S Mansor, Eileen R Gonzalez, Mark A Cole, Damian J Tyler, Jessica H Beeson, Kieran Clarke, Carolyn A Carr, Lisa C Heather

**Affiliations:** 1 Department of Physiology, Anatomy and Genetics, University of Oxford, Parks Road, OX1 3PT, Oxford, UK; 2 University of Nottingham Medical School, Queens Medical Centre, Nottingham, UK

## Abstract

**Background:**

To study the pathogenesis of diabetic cardiomyopathy, reliable animal models of type 2 diabetes are required. Physiologically relevant rodent models are needed, which not only replicate the human pathology but also mimic the disease process. Here we characterised cardiac metabolic abnormalities, and investigated the optimal experimental approach for inducing disease, in a new model of type 2 diabetes.

**Methods and results:**

Male Wistar rats were fed a high-fat diet for three weeks, with a single intraperitoneal injection of low dose streptozotocin (STZ) after fourteen days at 15, 20, 25 or 30 mg/kg body weight. Compared with chow-fed or high-fat diet fed control rats, a high-fat diet in combination with doses of 15–25 mg/kg STZ did not change insulin concentrations and rats maintained body weight. In contrast, 30 mg/kg STZ induced hypoinsulinaemia, hyperketonaemia and weight loss. There was a dose-dependent increase in blood glucose and plasma lipids with increasing concentrations of STZ. Cardiac and hepatic triglycerides were increased by all doses of STZ, in contrast, cardiac glycogen concentrations increased in a dose-dependent manner with increasing STZ concentrations. Cardiac glucose transporter 4 protein levels were decreased, whereas fatty acid metabolism-regulated proteins, including uncoupling protein 3 and pyruvate dehydrogenase (PDH) kinase 4, were increased with increasing doses of STZ. Cardiac PDH activity displayed a dose-dependent relationship between enzyme activity and STZ concentration. Cardiac insulin-stimulated glycolytic rates were decreased by 17% in 15 mg/kg STZ high-fat fed diabetic rats compared with control rats, with no effect on cardiac contractile function.

**Conclusions:**

High-fat feeding in combination with a low dose of STZ induced cardiac metabolic changes that mirror the decrease in glucose metabolism and increase in fat metabolism in diabetic patients. While low doses of 15–25 mg/kg STZ induced a type 2 diabetic phenotype, higher doses more closely recapitulated type 1 diabetes, demonstrating that the severity of diabetes can be modified according to the requirements of the study.

## Introduction

The incidence of type 2 diabetes continues to increase, despite the best current therapies and educational programs available. Cardiovascular disease is the leading cause of mortality in type 2 diabetic patients in the United Kingdom [1]. Metabolic changes in the heart have been implicated in the increased incidence of myocardial infarction [2], with diabetic patients having decreased cardiac glucose metabolism and increased cardiac fatty acid metabolism [3-6]. Therefore, a greater understanding of how type 2 diabetes affects the heart, the role of abnormal cardiac metabolism and how novel interventions could circumvent this are needed.

Animal models of type 2 diabetes are currently the first line for investigating disease mechanisms and pharmacological therapies. For relevance to humans, animal models must replicate the phenotype seen in patients as closely as possible, but it is also desirable that they mimic the developmental process of the disease. From a practical perspective, models that are easy to generate, cheap and develop in a timely manner will be favoured over expensive and time-consuming models. No animal models are perfect, and current rodent models of type 2 diabetes have been associated with a number of drawbacks (comprehensively reviewed by Bugger and Abel [7]). For example, the *db/db* mouse, *ob/ob* mouse and Zucker fatty rat have been extensively studied in the literature [8-11], and are generated by genetic abnormalities in the leptin signalling pathway, whereas, in patients, type 2 diabetes usually results as a consequence of multiple gene polymorphisms in combination with environmental factors. Similarly, the Goto-Kakizaki GK rat is insulin resistant but remains lean [12,13], making comparisons to the human condition and its association with obesity difficult. Another drawback of spontaneously diabetic and transgenic animals is the expensive cost of purchase [14]. In relation to cardiac research, one of the limitations of current rat models is the extended periods taken to develop cardiac phenotypes, despite the presence of abnormal circulating metabolites from an early age. For example, Zucker diabetic rats only show cardiac metabolic dysfunction after 12 weeks of age [15], and Zucker fatty rats only after 12 months [16]. Similarly, high-fat diet alone isn’t effective at modifying cardiac and systemic metabolism unless fed over an extended period [17].

A relatively new rat model was proposed first by Reed *et al.*[18], with modifications by Srinivasan *et al.*[19], which aimed to induce type 2 diabetes by using high-fat feeding to induce peripheral insulin resistance, followed by a low dose of the pancreatic β-cell toxin, streptozotocin (STZ). STZ is traditionally used at high doses to induce type 1 diabetes, as it results in impaired insulin secretion from the β-cell [20,21]. Reed *et al.* proposed that if a low dose of STZ was used after high-fat feeding, the function of the β-cell mass would be modestly impaired without completely compromising insulin secretion, resulting in a moderate impairment in glucose tolerance [18,19]. This would mimic the human disease process resulting in a metabolic phenotype similar to that in late stage type 2 diabetic patients. This model has become increasingly popular in recent years, both for investigating the mechanisms involved in type 2 diabetes and for testing potential therapies [22-26]. However, the degree of diabetes induced, the amount of STZ used, background strain and starting body weight vary considerably between these studies. As examples, Reed *et al.* administered 50 mg/kg STZ via an intravenous route following anaesthesia, Srinivasan *et al.* used 35 mg/kg STZ administered intraperitoneally but using relatively juvenile rats, whereas Zhang *et al.* fed rats on a high-fat diet for 2 months prior to STZ [18,19,27]. Therefore, a better understanding of the cardiac phenotype of this model, the metabolic changes associated with this method of inducing diabetes and determining the optimal protocol would be desirable prior to use in large scale studies.

Therefore, we set out to determine whether this high-fat feeding/low dose STZ model of type 2 diabetes modified cardiac metabolism in a similar manner to the human disease. In addition, we aimed to determine the optimal experimental approach to induce the disease by testing the cardiac effects of a variety of STZ doses in mature adult Wistar rats. Rats were fed a high-fat diet for three weeks, with a single intraperitoneal injection of STZ of either 15, 20, 25 or 30 mg/kg body weight after two weeks. Our results showed that inducing type 2 diabetes, using a combination of high-fat feeding with a low dose of STZ, mimics the human condition. We also demonstrate that while low doses of STZ induced type 2 diabetes and cardiac metabolic changes, a dose of 30 mg/kg induced overt and severe systemic alterations that more closely resembled type 1 diabetes.

## Methods

### Rat model of type 2 diabetes

Male Wistar rats (n = 55, 260 ± 7 g) were obtained from a commercial breeder (Harlan, UK). All procedures were in accordance with Home Office (UK) guidelines under The Animals (Scientific Procedures) Act, 1986 and with institutional guidelines. Control rats were fed for three weeks on a standard chow diet (Harlan Laboratories), with an Atwater Fuel Energy of 3.0 kcal/g, comprising 66% calories from carbohydrate, 22% from protein and 12% from fat (Additional file 1: Table S1). To induce diabetes, rats were fed a high-fat diet (Special Diet Services) for three weeks, with an Atwater Fuel Energy of 5.3 kcal/g, comprising 60% calories from fat, 35% from protein and 5% from carbohydrate, according to a modification of the protocols of Reed *et al.* and Srinivasan *et al.*[18,19]. On day 13, rats were fasted overnight and given a single intraperitoneal injection of streptozotocin (STZ in citrate buffer, pH 4) the following morning, and the high-fat diet feeding was continued for a further week (or chow diet for controls). Different doses of STZ (0, 15, 20, 25 and 30 mg/kg bodyweight *w/w*) in combination with high-fat diet, were investigated to determine the optimal dose to induce a type 2 diabetic phenotype with modified cardiac metabolism. We started our study with a dose of 30 mg/kg, to closely replicate that used by others [19], then included additional groups on lower doses of STZ until hyperglycaemia was no longer induced, mortality was not observed with any dose of STZ. After three weeks on their designated diet, rats in the fed state were terminally anaesthetised with sodium pentobarbital, hearts and livers were rapidly excised, freeze clamped and stored at −80°C for subsequent analysis. Following excision of the heart, blood was collected from the chest cavity, plasma separated and analysed for metabolites using a Pentra analyser (ABX, UK) and an insulin ELISA (Mercodia, Sweden). Both left and right epididymal fat pads were excised, trimmed and weighed, for assessment of adiposity.

### Tissue analysis

Cardiac and hepatic glycogen content were determined by the breakdown of glycogen to glucose units, using amyloglucosidase. Triglyceride content was measured in cardiac and hepatic tissue, following Folch extraction, using a kit from Randox, UK. The active fraction of pyruvate dehydrogenase was assayed in cardiac homogenates according to the protocol of Seymour *et al.*[28]. Medium chain acyl-coenzyme A dehydrogenase activity in cardiac homogenates was measured by following the decrease in ferricinium ion absorbance, as described by Lehman *et al.*[29]. Citrate synthase activity was measured in cardiac homogenates according to the method of Srere [30].

### Western blotting

Cardiac lysates were prepared from frozen tissue and equal concentrations of protein were loaded and separated on 12.5% SDS-PAGE gels, and transferred onto immobilon-p membranes (Millipore, UK) [31]. FAT/CD36 was detected with an antibody kindly donated by Dr Narendra Tandon (Otsuka Maryland Medicinal Laboratories, USA) [32]. Prof. Geoff Holman (University of Bath, UK) kindly donated the GLUT4 antibody [33,34]. PDK4 was detected using an antibody kindly donated by Prof. Mary Sugden (Queen Mary’s, University of London, UK) [35]. Antibodies against GLUT1 and UCP3 were purchased from Abcam, UK, and against monocarboxylate transporter (MCT) 1 from Santa Cruz. Even protein loading and transfer were confirmed by Ponceau staining.

### Isolated heart perfusion

A second group of rats were treated with the lowest dose of STZ (15 mg/kg) in combination with high-fat diet, to investigate the effect on cardiac glycolytic flux in the isolated perfused heart. Hearts were isolated into ice-cold Krebs-Henseleit (KH) buffer, cannulated via the aorta and perfused in Langendorff mode at a constant perfusion pressure of 100 mmHg at 37°C [31]. Hearts were perfused with 200 ml recirculating KH buffer (118 mM NaCl, 4.7 mM KCl, 1.2 mM MgSO_4_, 1.3 mM CaCl_2_, 0.5 mM EDTA, 25 mM NaHCO_3_, 1.2 mM KH_2_PO_4_, pH 7.4) containing 11 mM glucose and 3 U/L insulin, gassed with 95% O_2_ and 5% CO_2._

To measure functional changes during the perfusion protocol, a fluid filled, PVC balloon was inserted into the left ventricle, inflated to achieve an end-diastolic pressure of 4 mmHg, and attached via a polyethylene tube to a bridge amplifier and PowerLab data acquisition system (ADInstruments, Oxfordshire, UK). Left ventricular developed pressure was determined as systolic pressure minus end-diastolic pressure. Rate pressure product was calculated as the product of developed pressure and heart rate. To measure glycolytic rates, the KH buffer was supplemented with 0.2 μCi.ml^-1^ [5-^3^H]-glucose and timed aliquots of perfusate were collected during the perfusion protocol. Glycolytic rates were determined from the conversion of ^3^H-glucose to ^3^H_2_O in the aliquots.

### Statistics

Results are presented as means ± SEM, and were considered significant at p < 0.05 (SPSS Statistics 18). Differences between all six groups (control, 0, 15, 20, 25 and 30 mg/kg STZ) were investigated using a one-way ANOVA with Tukey post-hoc correction for multiple comparisons. To investigate the STZ dose-dependent effects, a two tailed regression analysis was performed between the five doses of STZ.

## Results

### Physical parameters

Control rats gained 69 ± 3 g in body weight over the three week protocol, averaging 23 ± 4 g per week (Table 1 and Additional file 2: Figure S1). Following the injection of STZ at day 14, the lower doses of STZ (0, 15, 20 and 25 mg/kg) did not alter total body weight gain or body weight gain in the final week compared with the control group. In contrast, the 30 mg/kg STZ dose induced weight loss in the final week despite the continuation of the high-fat diet, compared with the control group and other lower doses of STZ. There was a significant correlation between body weight gain in the final week and STZ doses (r^2^ = 0.33, p < 0.05). Epididymal fat pad weight, an indicator of total body adiposity, and fat pad to body weight ratio were significantly increased with higher doses of STZ compared with controls. Heart weight and heart weight to body weight ratio were not significantly different between any groups, demonstrating no significant cardiac hypertrophy.

**Table 1:** Physical parameters from control and diabetic rats, induced using high-fat feeding in combination with low dose STZ

		**Diabetes model**				
		**(High-fat feeding with low dose STZ)**				
	**Control**	**0 mg/kg**	**15 mg/kg**	**20 mg/kg**	**25 mg/kg**	**30 mg/kg**
Total body weight gain (g)	69 ± 3	82 ± 7	88 ± 15	93 ± 5	77 ± 9	50 ± 9 #
Body weight change during two weeks of diet modification (g)	46 ± 1	49 ± 7	56 ± 12	60 ± 2	55 ± 8	55 ± 7
Body weight change in final week following STZ or control (g)	23 ± 4	33 ± 5	32 ± 2	33 ± 3	22 ± 4	−5 ± 4 *†
Epidydimal fat pad weight (g)	4.8 ± 0.4	7.0 ± 0.7	5.7 ± 0.4	7.6 ± 0.7	9.8 ± 0.6 *#	8.3 ± 0.7 *
Epidydimal fat pad to body weight ratio (%)	1.5 ± 0.1	2.0 ± 0.2	1.8 ± 0.1	2.4 ± 0.2 *	2.5 ± 0.1 *	2.3 ± 0.2 *
Heart weight (g)	1.2 ± 0.1	1.2 ± 0.1	1.1 ± 0.1	1.1 ± 0.1	1.0 ± 0.1	1.1 ± 0.1
Heart to body weight ratio (× 10^3^)	3.6 ± 0.2	3.5 ± 0.1	3.4 ± 0.2	3.4 ± 0.2	3.2 ± 0.2	3.1 ± 0.1

* p < 0.05 vs. control, † p < 0.05 vs. all other doses of STZ, # p < 0.05 vs. 15 mg/kg STZ. n = 11 for control groups, n = 4 for diabetic groups.

### Plasma metabolites in the fed state

Blood glucose concentrations were not increased by high-fat diet alone or in combination with 15 mg/kg STZ, compared with control chow fed rats (Table 2). In contrast, blood glucose levels were increased by 20, 25 and 30 mg/kg STZ in combination with a high-fat diet, with the 30 mg/kg STZ dose increasing blood glucose significantly higher than all other groups. Plasma insulin concentrations were not significantly different to control animals at lower doses of STZ, but were significantly decreased by 30 mg/kg STZ. Non-esterified fatty acids (NEFA), triglyceride, and β-hydroxybutyrate (β-OHB) concentrations were increased only with the highest dose of STZ (30 mg/kg), compared with controls and the other doses of STZ investigated. Cholesterol concentrations were elevated by administration of 25 mg/kg STZ in combination with high-fat feeding compared with controls and lower doses of STZ. Regression analysis demonstrated significant positive relationships between STZ dose and plasma glucose (r^2^ = 0.37), triglycerides (r^2^ = 0.30), β-OHB (r^2^ = 0.41) and cholesterol (r^2^ = 0.20), and significant negative relationships between STZ dose and plasma insulin (r^2^ = 0.25) (p < 0.05 for all). Thus, while lower doses of 20–25 mg/kg STZ were sufficient to elevate blood glucose levels without impairing insulin secretion, the higher dose of STZ caused severe diabetes, as shown by increased glucose, β-OHB, triglycerides and decreased insulin concentrations.

**Table 2:** Plasma metabolites from control and diabetic rats in the fed state, induced using high-fat feeding in combination with low dose STZ

		**Diabetes model**				
		**(High-fat feeding with low dose STZ)**				
	**Control**	**0 mg/kg**	**15 mg/kg**	**20 mg/kg**	**25 mg/kg**	**30 mg/kg**
Glucose (mmol/l)	12.7 ± 0.3	14.6 ± 1.8	14.7 ± 0.6	17.2 ± 1.2 *	16.9 ± 1.5 *	25.1 ± 1.1 *†
Insulin (ug/l)	3.03 ± 0.51	2.68 ± 0.57	2.00 ± 0.31	1.59 ± 0.36	1.93 ± 0.26	1.04 ± 0.22 *
NEFA (mmol/l)	0.28 ± 0.06	0.29 ± 0.05	0.29 ± 0.02	0.29 ± 0.01	0.14 ± 0.04	0.59 ± 0.07 *#
Triglycerides (mmol/l)	1.79 ± 0.20	1.13 ± 0.22	0.87 ± 0.06	1.41 ± 0.15	1.79 ± 0.35	7.25 ± 1.33 *†
β-OHB (mmol/l)	0.26 ± 0.04	0.33 ± 0.04	0.45 ± 0.07	0.77 ± 0.06	0.98 ± 0.21	5.39 ± 0.59 *†
Cholesterol (mmol/l)	2.46 ± 0.13	2.07 ± 0.21	1.68 ± 0.15	2.11 ± 0.27	3.87 ± 0.58 *$	3.02 ± 0.44 €

* p < 0.05 vs. control, † p < 0.05 vs. all other doses of STZ, # p < 0.05 vs. 15 and 25 mg/kg, $ p < 0.05 vs. 0, 15 and 20 mg/kg, € p < 0.05 vs. 15 mg/kg. *NEFA* non-esterified fatty acids, *β-OHB* β-hydroxybutyrate, control n = 11, for diabetic groups n = 4.

### Hepatic intracellular substrate stores

**Figure 1:**
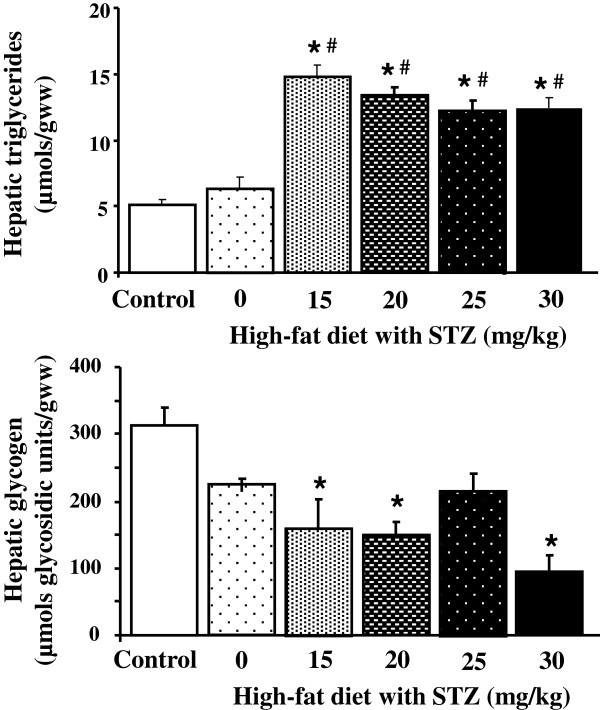
**Hepatic triglyceride and glycogen concentrations in control and diabetic rats following high-fat feeding in combination with low dose STZ.** * p < 0.05 vs. control, # p < 0.05 vs. high-fat only, n = 10 for control group, n = 4 for diabetic groups.

Hepatic triglyceride and glycogen stores were measured as an indicator of changes in liver metabolism. Three weeks of high-fat diet alone did not change hepatic triglyceride or glycogen concentrations compared with controls (Figure 1). Combining high-fat diet with all doses of STZ tested increased hepatic triglyceride concentrations, compared with control rat livers. In contrast, hepatic glycogen concentrations were decreased following induction of diabetes using high-fat feeding in combination with STZ at doses of 15, 20 and 30 mg/kg STZ. Both measurements correlated with STZ dose (triglycerides r^2^ = 0.39, glycogen r^2^ = 0.23, p < 0.05).

### Cardiac intracellular substrate stores

**Figure 2:**
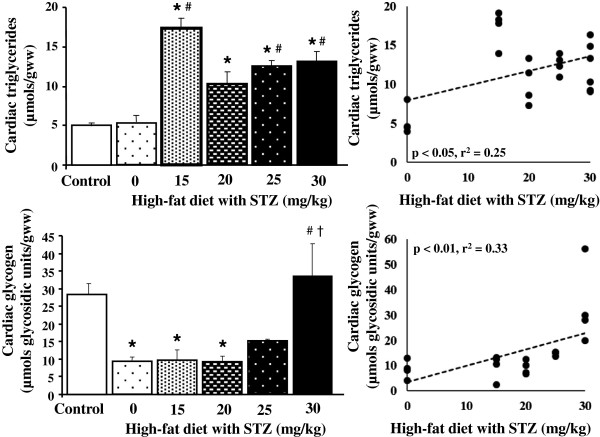
**Cardiac triglyceride and glycogen concentrations in control and diabetic rats following high-fat feeding in combination with low dose STZ.** * p < 0.05 vs. control, # p < 0.05 vs. high-fat only, † p < 0.05 vs. 15 and 20 mg/kg STZ, n = 9 for control group, n = 4 for diabetic groups.

One of the main aims of this study was to characterise the cardiac metabolic phenotype of this new model of type 2 diabetes. Cardiac triglyceride concentrations were increased by all doses of STZ tested, compared with control rats, but not by high-fat diet alone, displaying a significant positive correlation with STZ dose (Figure 2). Cardiac glycogen concentrations were decreased by high-fat diet and lower doses of STZ (15 and 20 mg/kg), but showed a dose-dependent increase with STZ concentration. The highest dose of STZ tested (30 mg/kg) did not change cardiac glycogen concentrations compared with controls, and was significantly higher than lower doses of STZ (15 and 20 mg/kg).

### Cardiac enzyme assays

**Figure 3:**
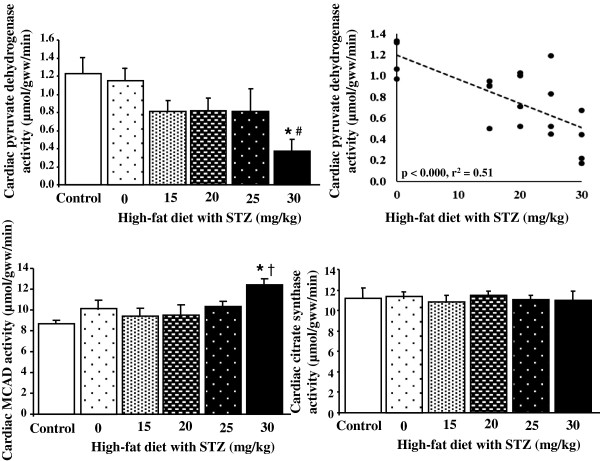
**Cardiac pyruvate dehydrogenase, medium chain acyl-coenzyme A dehydrogenase (MCAD) and citrate synthase activities in control and diabetic rats following high-fat feeding in combination with low dose STZ.** * p < 0.05 vs. control, # p < 0.05 vs. high-fat only, † p < 0.05 vs. 15 and 20 mg/kg STZ, n = 4–5 per group.

Cardiac pyruvate dehydrogenase (PDH) activity, a heavily regulated enzyme of mitochondrial glucose metabolism, was significantly decreased in diabetic hearts at a dose of 30 mg/kg STZ, compared with controls (Figure 3). There was a significant negative correlation between PDH activity and STZ dose. In contrast, medium chain acyl-coenzyme A dehydrogenase (MCAD) activity, an enzyme involved in fatty acid β-oxidation, was significantly increased in 30 mg/kg STZ diabetic rats, compared with controls. These changes in PDH and MCAD activity were independent of a change in the Krebs cycle enzyme citrate synthase, used as a marker of overall mitochondrial content.

### Cardiac metabolic proteins

**Figure 4:**
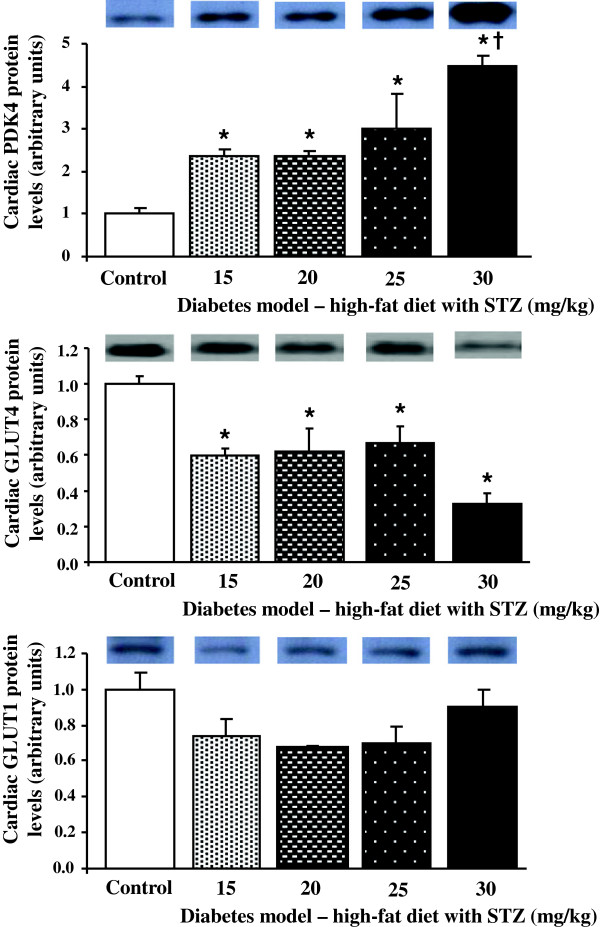
**Cardiac pyruvate dehydrogenase kinase 4 (PDK4), glucose transporters (GLUT) 4 and 1 protein levels in control and diabetic rats following high-fat feeding in combination with low dose STZ.** * p < 0.05 vs. control, † p < 0.05 vs. all other doses of STZ, n = 6 for control group, n = 4 for diabetic groups.

**Figure 5:**
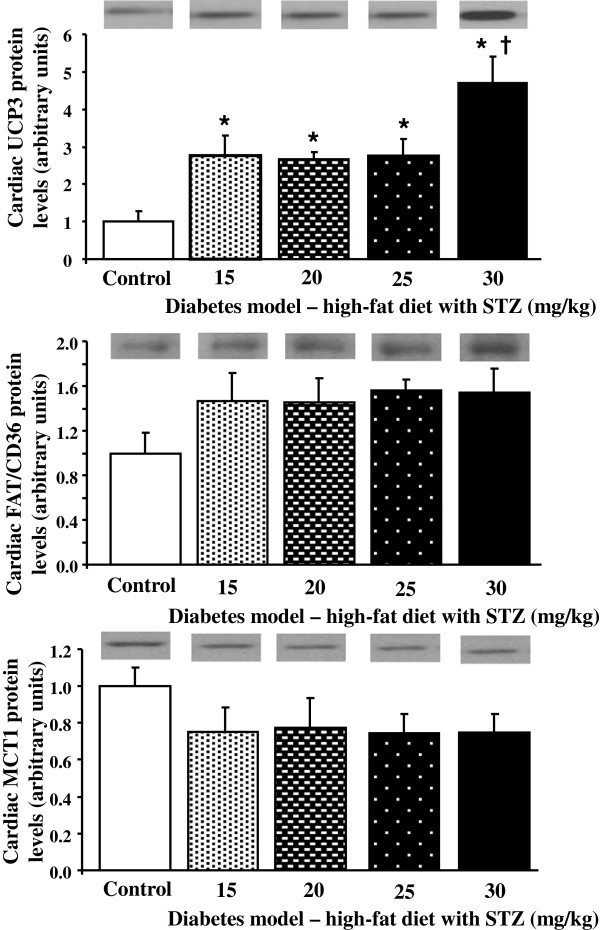
**Cardiac uncoupling protein 3 (UCP3), fatty acid translocase (FAT/CD36) and monocarboxylate transporter 1 (MCT1) protein levels in control and diabetic rats following high-fat feeding in combination with low dose STZ.** * p < 0.05 vs. control, † p < 0.05 vs. all other doses of STZ, n = 6 for control group, n = 4 for diabetic groups.

As the changes in metabolic enzymes displayed dose-dependent relationships between STZ dose and activities, we, therefore, investigated which doses of STZ induced changes in metabolic protein expression. Proteins involved in cardiac glucose metabolism were investigated in our model of type 2 diabetes, to determine if this pathway was downregulated in diabetic hearts (Figure 4). Protein levels of the PDH inhibitor pyruvate dehydrogenase kinase 4 (PDK4), were increased by all doses of STZ compared with controls. In contrast, the insulin-responsive glucose transporter, GLUT4, protein levels decreased with all doses of STZ, compared with control hearts. Both PDK4 and GLUT4 displayed dose-dependent relationships with STZ concentrations (r^2^ = 0.53 and r^2^ = 0.22, respectively, p < 0.05). GLUT1 protein levels showed no significant differences between groups. Markers of cardiac fatty acid metabolism were also assessed, to determine if this pathway was upregulated in our diabetic hearts (Figure 5). Uncoupling protein 3 (UCP3), a fatty acid regulated protein, was increased by all doses of STZ compared with control hearts, increasing in a dose-dependent manner with STZ concentration (r^2^ = 0.34, p < 0.05). FAT/CD36, a fatty acid transporter, was not significantly different between groups when assessed by one way ANOVA. MCT1, responsible for ketone body and monocarboxylic acid uptake, was measured, to determine if changes in this transporter mirrored changes in plasma ketone bodies. There were no significant differences between groups in cardiac MCT1 protein levels, although there was a general trend for lower levels in diabetic hearts compared with control hearts.

### Cardiac glycolytic rates from 15 mg/kg STZ high-fat fed diabetic rats

**Figure 6:**
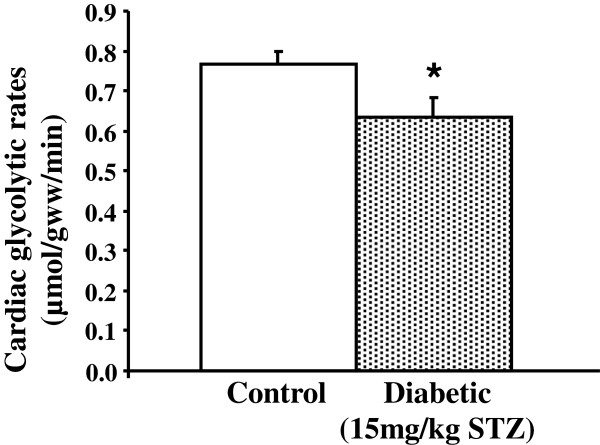
**Glycolytic rates in isolated perfused hearts from control and 15 mg/kg STZ in combination with high-fat fed diabetic rats.** * p < 0.05 vs. control, n = 11 for control, n = 6 for 15 mg/kg diabetics.

All doses of STZ investigated showed a downregulation of glucose metabolism proteins, therefore, we questioned whether this was sufficient to affect flux through the glycolytic pathway in the perfused, contracting heart. Given that a number of metabolic changes in these hearts displayed a dose-dependent relationship, we rationalised that if we saw a change in glycolysis with the lowest dose (15 mg/kg STZ), then this would likely indicate that overall the model was sufficient to inhibit cardiac glucose metabolic flux (Figure 6).

Hearts from 15 mg/kg STZ high-fat fed diabetic rats did not show any defects in contractile function. Heart rates (284 ± 13 and 264 ± 5 bpm in control and diabetic hearts, respectively), developed pressures (165 ± 10 and 182 ± 10 mmHg in control and diabetic hearts, respectively), and rate pressure products (47 ± 4 and 48 ± 3 × 10^3^ mmHg/min in control and diabetic hearts, respectively) were not significantly different between control and 15 mg/kg STZ diabetic hearts. In contrast, glycolytic rates in the presence of insulin were significantly decreased by 17% in 15 mg/kg type 2 diabetic rat hearts compared with control hearts. Thus, even at the lowest dose of STZ, glycolytic rates were suppressed in the hearts of these type 2 diabetic rats.

## Discussion

This study has demonstrated that high-fat feeding in combination with a low dose of STZ is sufficient to induce the cardiac metabolic phenotype present in type 2 diabetes. In general, this model displayed hyperglycaemia, normoinsulinaemia and hepatic lipid deposition. Diabetic hearts had decreased proteins involved in glucose metabolism with a concomitant increase in proteins involved in fat metabolism. Many metabolic parameters displayed a dose-dependent relationship with STZ, with the highest dose of STZ inducing a metabolic profile that more closely resembled type 1 diabetes. Therefore, this model of type 2 diabetes would appear to mirror the human condition, but care must be taken when determining the dose of STZ to use and the subsequent degree of diabetes induced.

The highest dose of STZ tested (30 mg/kg) induced systemic changes that more closely resembled the type 1 diabetic phenotype. Only this high dose of STZ induced weight loss, and produced a plasma metabolite profile that included hyperketonaemia, hyperlipidaemia, and hypoinsulinaemia. This is in agreement with other studies that have used high doses of STZ in isolation or in combination with high-fat feeding [19,36-39], with these high doses also causing abnormalities in liver morphology and function [25]. In contrast, the lower doses of STZ investigated in the present study avoided these extreme phenotypes, instead presenting with hyperglycaemia in the absence of ketosis, normoinsulinaemia and with maintenance of body weight. Increased adiposity relative to body weight was observed with doses of 20, 25 and 30 mg/kg STZ, and wasn’t present with high-fat diet only, demonstrating a combined effect of the dietary manipulation and STZ administration. The increased adiposity and elevated cholesterol induced by 25 mg/kg STZ are features present in other models of type 2 diabetes [40-42], suggesting that, from a systemic point of view, the middle range dose of STZ tested was the most desirable for future work.

In patients with type 2 diabetes, non-invasive imaging studies have demonstrated a metabolic shift in cardiac substrate metabolism [3-6], with glucose metabolism suppressed and fatty acids metabolism elevated, characteristic of the Randle cycle [43]. This metabolic shift has been demonstrated in a number of animal models of diabetic cardiomyopathy [44,45], and has been implicated in the increased incidence of, and decreased recovery following, myocardial infarction [2]. In our diabetic hearts, we found markers of fatty acid oxidation, such as UCP3, were upregulated. UCP3, MCAD and PDK4 are peroxisome proliferator-activated receptor α (PPARα) targets, a transcription factor that is activated by fatty acid ligands to upregulate fat metabolism, and is increased in diabetes [46]. The activation of PPARα in diabetes is due to both the increased intake of dietary fatty acids, in addition to increased adipose lipolysis associated with adipose insulin resistance [47]. In contrast to fatty acid metabolism, proteins involved in cardiac glucose metabolism, such as GLUT4, were downregulated in our diabetic hearts. Certainly, the large increase in PDK4 with 30 mg/kg STZ, would account for the decrease in PDH activity in these hearts via inhibitory phosphorylation of this complex. Thus, the changes in protein and enzyme activity in our diabetic hearts would fully support the shift away from glucose metabolism towards fat metabolism, reported in other models and in patient studies [48]. Future studies to measure the effects on fatty acid and oxidative metabolism in this model will confirm the link between the changes in mitochondrial proteins and flux through these pathways.

Perfused heart studies allow the simultaneous measurement of metabolic flux and contractile function in the isolated organ. Even at the lowest dose of STZ, glycolytic rates were decreased, confirming that our changes in proteins were sufficient to impact on overall flux through the pathway, in agreement with studies on *db/db* and *ob/ob* mice [44,45,49,50]. The decrease in glycolysis was independent of impaired cardiac systolic function or loss of mitochondria, suggesting that the glycolytic changes were not secondary to adverse cardiac remodelling and mitophagy. In a clever study by Marsh *et al.*, the interaction between diet and diabetes on cardiovascular function was investigated, using a similar model to our current study [51]. They demonstrated that a combination of high-fat feeding and low dose STZ increased diastolic wall stress and arterial stiffness, as occurs in patients with type 2 diabetes, but that modifying only diet or using only STZ did not produce this effect, despite increased blood glucose and abnormal insulin tolerance tests, respectively [51]. Thus, inducing type 2 diabetes using high-fat feeding and low dose STZ, not only mimics the cardiac metabolic phenotype but also replicates the diastolic dysfunction and vascular complications associated with the human disease.

Overall, these data support the use of the high-fat diet/low dose STZ approach in the development of a type 2 diabetic model for future cardiac studies. The advantages of this model are that the disease is induced over a relatively short time and without high costs. In addition, the dose of STZ can be manipulated to match the degree of diabetes required for the study. In a study by Watts *et al*., this model of high-fat diet and low dose STZ was used and directly compared to the ZDF rat, with both models demonstrating the same hepatic and adipose effects, but the errors associated with the ZDF animals were much greater than with the high-fat/STZ model, suggesting that the reproducibility may be improved by using this new model [22]. Similarly, in a study by Islam and Choi, the high-fat diet in combination with low dose STZ model was identified as a better model of type 2 diabetes than an alternative chemically-induced model that utilised an injection of nicotinamide prior to administration of STZ [37]. We found it was essential to fast the rats prior to STZ injection, as preliminary experiments demonstrated a much lower success rate for inducing diabetes if injections were carried out in the fed state (data not shown). This is likely related to the mode of action of STZ, a glucosamine-nitrosourea antibiotic that competes with blood glucose for the pancreatic β-cell GLUT2 receptor [20].

Hepatic and cardiac triglyceride concentrations were elevated by all doses of STZ tested but not by high-fat diet alone, indicating that this was due to the combination of STZ and high-fat. Hepatic and cardiac glycogen concentrations showed dose-dependent relationships with STZ concentrations, but in opposite directions in the two organs; decreasing in liver but increasing in heart. Interestingly, hepatic glycogen was not affected by high-fat diet alone, in contrast, cardiac glycogen was significantly decreased just by the presence of high-fat diet, demonstrating different regulation of glycogen deposition in these two organs.

From our data, doses of 30 mg/kg STZ (and potentially above) would be less desirable than lower doses for modelling type 2 diabetes, due to the extreme systemic phenotype induced. However, at no time did we see an increase in fed insulin concentrations, which has been observed in a number of other models. It has been suggested that this is due to STZ causing a small degree of β-cell damage, which is sufficient to limit the upregulation of insulin secretion in response to the systemic insulin resistance [19]. Srinivasan *et al.* demonstrated that 35 mg/kg STZ had no effect on insulin concentrations in chow fed rats, whereas high-fat feeding in isolation increased insulin, with the combination of high-fat and STZ bringing insulin concentrations back to control levels [19]. Using a glucose tolerance test, they demonstrated systemic insulin resistance with high-fat diet alone [19]. Thus, it could be that our model using 30 mg/kg STZ mimics a later stage in type 2 diabetes-insulin resistance disease progression, when β-cell function starts to become compromised and no longer matches the increased demand for insulin.

In conclusion, a combination of high-fat feeding with a low dose of STZ provides a model of type 2 diabetes that mimics the metabolic phenotype present in patients. This model also has the added advantages of being relatively inexpensive, easy to induce and can be modified for different severities of diabetes, according to requirement of the study. For future studies we would use a dose of 25 mg/kg STZ in combination with high-fat feeding, as this induced adiposity, hypercholesterolemia, mild hyperglycaemia without compromising insulin secretion, and exhibited cardiac metabolic changes that mirrored the well characterised shift from glucose to fatty acid metabolism in type 2 diabetes.

## Abbreviations

STZ: Streptozotocin; FAT/CD36: Fatty acid translocase; GLUT: Glucose transporter; PDH: Pyruvate dehydrogenase; MCAD: Medium chain acyl-coenzyme A dehydrogenase; PDK: Pyruvate dehydrogenase kinase; UCP: Uncoupling protein.

## Competing interests

The authors declare that they have no competing interests.

## Authors’ contributions

LM conducted the isolated heart perfusion experiments, enzyme assays and tissue analysis. EG and JB conducted the enzyme assays and western blotting experiments. MC, DT, CC and KC conducted the animal experiments, assisted with data analysis and helped to draft the manuscript. LH performed experiments, carried out the statistical analysis and drafted the manuscript. All authors read and approved the final manuscript.

## Supplementary Material

Additional file 1: Table S1 Composition of chow and high-fat diet. Click here for file

Additional file 2: Figure S1 Body weight gain in control and diabetic rats. Rats were fed a chow or high-fat diet for 21 days, with STZ injected at varying doses at day 14. n = 4–11 per group. Click here for file
