# Supplementary material for: Cardiac metabolism in a new rat model of type 2 diabetes using high-fat diet with low dose streptozotocin
Source: Cardiovasc Diabetol. 2013 Sep 24;12:136. doi: 10.1186/1475-2840-12-136 (PMC3849358; doi:10.1186/1475-2840-12-136)
Supplement: Additional file 1: Table S1 — Composition of chow and high-fat diet. [file 1475-2840-12-136-S1.ppt]

## Slide 1
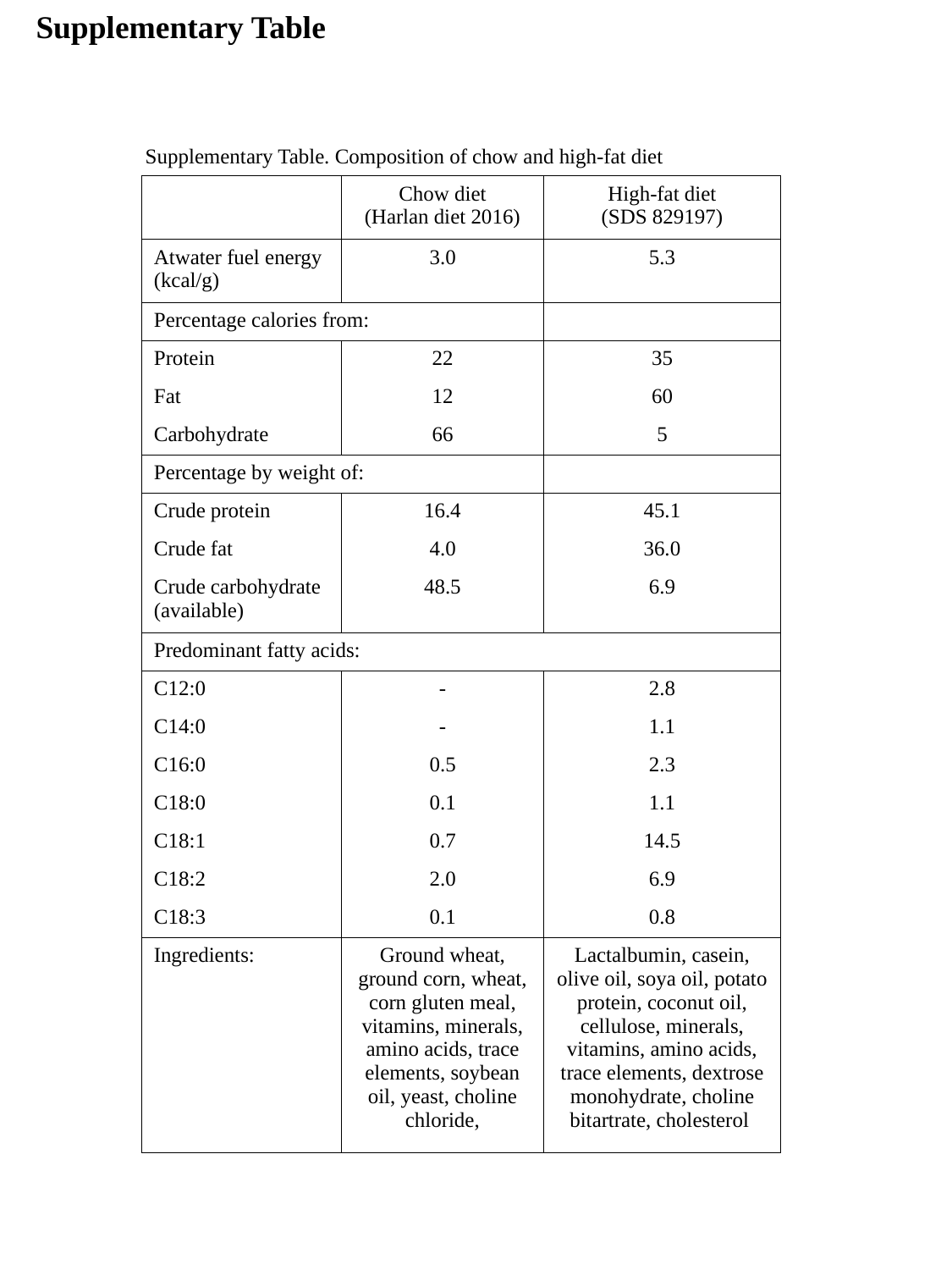

Supplementary Table
Supplementary Table. Composition of chow and high-fat diet
| | Chow diet (Harlan diet 2016) | High-fat diet (SDS 829197) |
| --- | --- | --- |
| Atwater fuel energy (kcal/g) | 3.0 | 5.3 |
| Percentage calories from: | | |
| Protein | 22 | 35 |
| Fat | 12 | 60 |
| Carbohydrate | 66 | 5 |
| Percentage by weight of: | | |
| Crude protein | 16.4 | 45.1 |
| Crude fat | 4.0 | 36.0 |
| Crude carbohydrate (available) | 48.5 | 6.9 |
| Predominant fatty acids: | | |
| C12:0 | - | 2.8 |
| C14:0 | - | 1.1 |
| C16:0 | 0.5 | 2.3 |
| C18:0 | 0.1 | 1.1 |
| C18:1 | 0.7 | 14.5 |
| C18:2 | 2.0 | 6.9 |
| C18:3 | 0.1 | 0.8 |
| Ingredients: | Ground wheat, ground corn, wheat, corn gluten meal, vitamins, minerals, amino acids, trace elements, soybean oil, yeast, choline chloride, | Lactalbumin, casein, olive oil, soya oil, potato protein, coconut oil, cellulose, minerals, vitamins, amino acids, trace elements, dextrose monohydrate, choline bitartrate, cholesterol |
