# Supplementary material for: Cardiac metabolism in a new rat model of type 2 diabetes using high-fat diet with low dose streptozotocin
Source: Cardiovasc Diabetol. 2013 Sep 24;12:136. doi: 10.1186/1475-2840-12-136 (PMC3849358; doi:10.1186/1475-2840-12-136)
Supplement: Additional file 2: Figure S1 — Body weight gain in control and diabetic rats. Rats were fed a chow or high-fat diet for 21 days, with STZ injected at varying doses at day 14. n = 4–11 per group. [file 1475-2840-12-136-S2.ppt]

## Slide 1
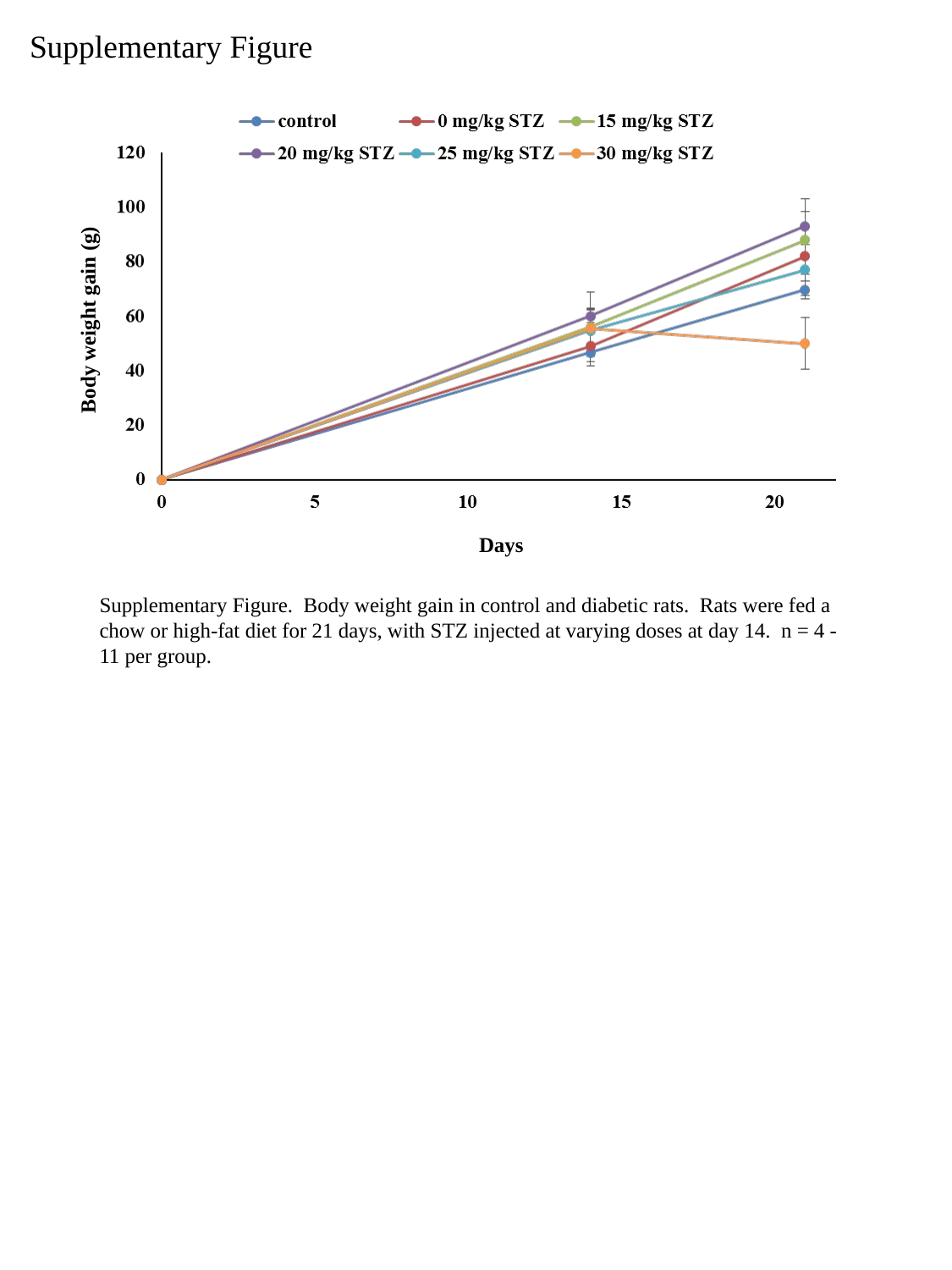

Supplementary Figure
Body weight gain (g)
Days
Supplementary Figure. Body weight gain in control and diabetic rats. Rats were fed a chow or high-fat diet for 21 days, with STZ injected at varying doses at day 14. n = 4 - 11 per group.
